# Kaplan’s Lesion in a Child: A Case Report

**DOI:** 10.31729/jnma.7892

**Published:** 2022-11-30

**Authors:** Abhishek Kumar Thakur, Nabees Man Singh Pradhan, Pramod Devkota, Bidur Gyawali, Prabhav Majgaiyan Pokhrel

**Affiliations:** 1 Department of Orthopedics and Trauma Surgery, Patan Academy of Health Sciences, Lagankhel, Lalitpur, Nepal

**Keywords:** *joint dislocations*, *metacarpophalangeal joint*, *open reduction*

## Abstract

Kaplan's lesion is a rare complex metacarpophalangeal joint dislocation. A 7-year-old female child presented with pain, swelling and inability to move her right index finger. Her mother gave a history of sustaining a fall injury on the same hand around 3 weeks back. Radiographs showed a complex dorsal metacarpophalangeal joint dislocation. As the injury was already 3 weeks old at presentation, a few attempts at closed reduction were tried, under anaesthesia, which was unsuccessful. So, the patient underwent open reduction through a dorsal approach. At a 1-year follow-up, the patient was pain-free and had regained full range of motion of the index finger metacarpophalangeal joint. The differential diagnosis of Kaplan's lesion should be considered when a child presents with finger dislocation.

## INTRODUCTION

Kaplan's lesion is a complex metacarpophalangeal (MCP) joint dislocation. It is uncommon in children and rarely reported.^1^ Kaplan first published his article describing the numerous anatomical interposing structures which prevent reduction by closed methods.^2^ MCP joint dislocations that cannot be reduced by closed methods and require open reduction are termed complex dislocations.^3^ There are two well-recognized surgical reduction approaches the dorsal and volar approaches.^4^ We report a case of Kaplan's lesion in a child.

## CASE REPORT

**Figure 1:**
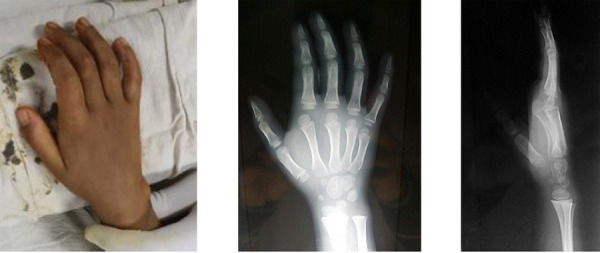
A) Pre-operative clinical picture, B) Preoperative X-ray images.

A 7-year-old female child presented to our OPD with pain, swelling and inability to move her right index finger for 3 weeks. Her mother mentioned that she had sustained a fall injury on her right hand while playing. The past, family and social history were insignificant. On examination, there was a hyperextension deformity at the second MCP joint with minimal swelling and tenderness. Both active and passive movements at the same joint were restricted. This gave an impression of a probable fracture or dislocation around the MCP or interphalangeal joint. X-ray radiographs showed a complex dorsal dislocation of the second MCP joint i.e. Kaplan's lesion (Figure 1).

**Figure 2:**
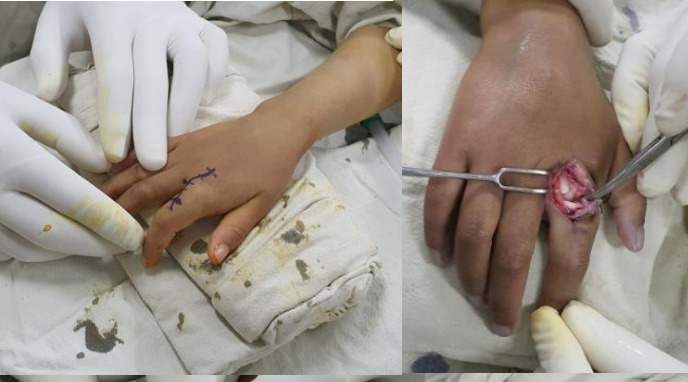
Intraoperative images.

Under general anaesthesia, a few attempts at closed reduction were tried which were unsuccessful and an open reduction was planned. The second MCP joint was approached dorsally. The extensor tendon and underlying capsule were split longitudinally to expose the volar plate which was incised longitudinally and reduction was achieved. A tiny osteochondral fragment from the metacarpal head was found which was fixed with an absorbable suture (Figure 2).

**Figure 3:**
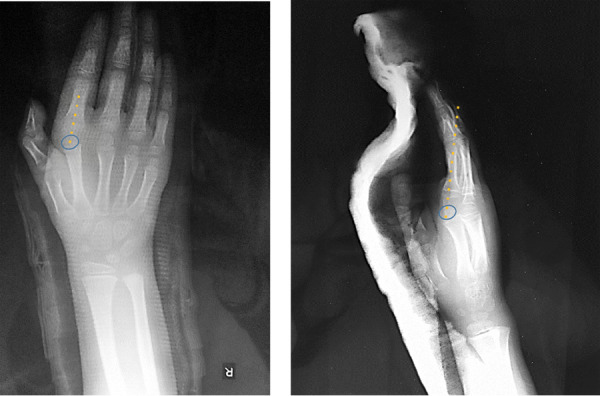
Postoperative X-Ray images.

Post-operative radiographs were taken to review the adequacy of reduction. A below elbow volar slab was kept for 3 weeks followed by active range of motion exercises (Figure 3).

At 1-year follow-up, the patient was pain-free and had regained full range of motion of the index finger MCP joint.

## DISCUSSION

MCP joint dislocations are relatively uncommon and occur less often than interphalangeal joint dislocations.^4^ A study done in India reported five cases in a 2-year time period in 2015.^2^ Another Indian study reported 20 cases in a period of 7 years in 2018. Kaplan's lesion is a rare injury.^5^ It is an underreported injury in Orthopaedic practice and so far, no cases have been reported from Nepal. In this case, the patient presented with pain, swelling and inability to move her index finger which was similar to a study done in India.^2,4^

Kaplan's original description clearly points out the pathoanatomy of MCP joint dislocation - the fibrocartilaginous plate avulses from its weakest attachment i.e. the volar aspect of the metacarpal neck with the flexor tendons and the pretendinous band displaced ulnarly and the lumbricals displaced radially to the metacarpal head. Closed reduction is often not successful as the flexor tendons, the pretendinous band and the lumbricals form a rigid constriction noose around the head, leading to the irreducibility of the dislocation.^5^ This is the reason for Kaplan's lesion requiring treatment and by open approach in our practice.

Two primary approaches have been described for open reduction - the dorsal and the volar. The dorsal approach carries less risk to the digital neurovascular bundle and the volar approach allows repair of the volar plate. A study from the USA reported using the dorsal approach for open reduction of Kaplan's lesion in a 7-year-old child.^4^ Similarly, another study in India used the dorsal approach in two and the volar approach in one child with MCP joint dislocation of thumb.^6^ The choice of approach depends on the surgeon's preference. We used a dorsal approach for an open reduction in our patient.

After open reduction, the MCP joint must be immobilised in a functional position for less than 3 weeks. Immobilisation for a longer period may cause early degenerative arthritis or decreased range of motion.^7^ Our patient had regained full range of motion of her index finger MCP joint and was very comfortable using her hand at 1-year follow-up. Her x-ray also did not reveal any abnormalities.

The patient and her mother were satisfied with the treatment outcome and her ability to return back to her usual activities. This case shows that finger dislocations in children can also present as Kaplan's lesion. Kaplan's lesion, a complex MCP joint dislocation, is a rare injury in adults and even rarer in children, with no similar case reports from Nepal. Late presentation necessitates open reduction. Open reduction by the dorsal approach has the advantage of lesser damage to digital neurovascular structures.
